# Tick sialostatins L and L2 differentially influence dendritic cell responses to *Borrelia* spirochetes

**DOI:** 10.1186/s13071-015-0887-1

**Published:** 2015-05-15

**Authors:** Jaroslava Lieskovská, Jana Páleníková, Helena Langhansová, Andrezza Campos Chagas, Eric Calvo, Michalis Kotsyfakis, Jan Kopecký

**Affiliations:** Faculty of Science, University of South Bohemia, Branišovská 1760, CZ-37005 České Budějovice, Czech Republic; Institute of Parasitology, Biology Centre of the Academy of Sciences of the Czech Republic, Branišovská 31, CZ-37005 České Budějovice, Czech Republic; Laboratory of Malaria and Vector Research, National Institute of Allergy and Infectious Diseases, National Institutes of Health, 12735 Twinbrook Parkway, Rockville, MD 20852 USA

**Keywords:** Dendritic cells, *Borrelia burgdorferi*, Tick cystatin, Signalling

## Abstract

**Background:**

Transmission of pathogens by ticks is greatly supported by tick saliva released during feeding. Dendritic cells (DC) act as immunological sentinels and interconnect the innate and adaptive immune system. They control polarization of the immune response towards Th1 or Th2 phenotype. We investigated whether salivary cystatins from the hard tick *Ixodes scapularis*, sialostatin L (Sialo L) and sialostatin L2 (Sialo L2), influence mouse dendritic cells exposed to *Borrelia burgdorferi* and relevant Toll-like receptor ligands.

**Methods:**

DCs derived from bone-marrow by GM-CSF or Flt-3 ligand, were activated with *Borrelia* spirochetes or TLR ligands in the presence of 3 μM Sialo L and 3 μM Sialo L2. Produced chemokines and IFN-β were measured by ELISA test. The activation of signalling pathways was tested by western blotting using specific antibodies. The maturation of DC was determined by measuring the surface expression of CD86 by flow cytometry.

**Results:**

We determined the effect of cystatins on the production of chemokines in *Borrelia*-infected bone-marrow derived DC. The production of MIP-1α was severely suppressed by both cystatins, while IP-10 was selectively inhibited only by Sialo L2. As TLR-2 is a major receptor activated by *Borrelia* spirochetes, we tested whether cystatins influence signalling pathways activated by TLR-2 ligand, lipoteichoic acid (LTA). Sialo L2 and weakly Sialo L attenuated the extracellular matrix-regulated kinase (Erk1/2) pathway. The activation of phosphatidylinositol-3 kinase (PI3K)/Akt pathway and nuclear factor-κB (NF-κB) was decreased only by Sialo L2. In response to *Borrelia burgdorferi*, the activation of Erk1/2 was impaired by Sialo L2. Production of IFN-β was analysed in plasmacytoid DC exposed to *Borrelia*, TLR-7, and TLR-9 ligands. Sialo L, in contrast to Sialo L2, decreased the production of IFN-β in pDC and also impaired the maturation of these cells.

**Conclusions:**

This study shows that DC responses to *Borrelia* spirochetes are affected by tick cystatins. Sialo L influences the maturation of DC thus having impact on adaptive immune response. Sialo L2 affects the production of chemokines potentially engaged in the development of inflammatory response. The impact of cystatins on *Borrelia* growth *in vivo* is discussed.

**Electronic supplementary material:**

The online version of this article (doi:10.1186/s13071-015-0887-1) contains supplementary material, which is available to authorized users.

## Background

*Borrelia burgdorferi*, the causative agent of Lyme disease, is transmitted to mammals through the bite of infected *Ixodes* ticks. In the skin, dendritic cells (DC) are among the first immune cells to come into contact with *B. burgdorferi* [1]. *B. burgdorferi* elicits a potent cytokine/chemokine response through activation of multiple pattern recognition receptors on innate immune cells, including Toll-like receptor (TLRs), NOD-like receptors (NLRs), and C-type lectin receptors (CLRs) [2]. TLRs have an essential role in the control of *B. burgdorferi* burden, because mice deficient in the common TLR signaling molecule myeloid differentiation primary response 88 (MyD88), have up to 250-fold more spirochetes than the wild-type controls [3, 4]. Among Toll-like receptors (TLRs), TLR-2 has been found to be the most important receptor for induction of pro-inflammatory mediators, whereas endosomal receptors TLR-7 and TLR-9 mediate type I interferon production [5–9]. All these TLRs utilize MyD88 as adaptor molecule, however, TLR-2 dependent inflammatory responses to *B. burgdorferi* can also be mediated by Toll-IL-1 receptor domain-containing adaptor inducing IFN-β (TRIF) [10]. *Borrelia* spirochetes activate multiple signalling pathways through these adaptors, including nuclear factor-κB (NF-κB), mitogen-activated protein kinases (MAPK) (extracellular matrix-regulated kinase (Erk) 1/2, p38, Janus N-terminal kinase (JNK)) [11–13], phosphatidylinositol-3 kinase (PI3K) [14], and Protein kinase C (PKC) pathways [15]. The p38 MAPK and NF-κB are critically involved in the expression of pro-inflammatory cytokines [12, 16], whereas PI3K pathway is fundamental for optimal phagocytosis [14]. *Borrelia* also strongly induces anti-inflammatory cytokine IL-10, which has overall suppressive effect on induction of pro-inflammatory mediators [17, 18].

Dendritic cells, as a part of innate immune system, produce several cytokines and chemokines which in autocrine and paracrine manner regulate the establishment of an innate immune response, including the recruitment of monocytes, macrophages, and neutrophils [19]. In addition, DC upon sensing pathogens undergo the maturation process, characterized by increased expression of co-stimulatory molecules, which is necessary for proper presentation of antigen to naïve T-cells. *In vitro*, dendritic cells can be obtained by culturing of bone- marrow cells in the presence of two cytokines, granulocyte-macrophage colony-stimulated factor (GM-CSF) or Fms-like tyrosine kinase 3 ligand (Flt-3L), respectively. By GM-CSF, the myeloid subset of dendritic cells can be generated, while with the Flt-3 ligand, lymphoid- type of plasmacytoid dendritic cells (pDC) can be obtained [20, 21]. The pDC are characterised by robust production of type I IFN [22]. These subsets of DC differ in the cytokine profiles they induce in T cells *in vivo* [23].

Dendritic cells are key players in host defense against tick-transmitted borreliae [1]. However, many functions of DC are negatively influenced by tick saliva [24–26]. In addition to prostaglandin E2 [27], purine nucleoside adenosine [28] and Salp15 [29], tick cystatins are also involved in the effect of tick saliva on dendritic cells [30].

Sialostatins L and L2 are cysteine protease inhibitors of the hard tick *Ixodes scapularis.* Both are strong inhibitors of cathepsin L [31, 32], but sialostatin L also inhibits cathepsin S. Immunosuppressive effects of Sialo L have been demonstrated in T cell line CTLL-2 [32] and lipopolysaccharide-activated DC [33]. Expression of Sialo L2 is greatly enhanced by feeding and is necessary for tick feeding success [34]. In addition to being able to enhance the growth of *Borrelia burgdorferi in vivo* [35], this sialostatin has been shown to inhibit the inflammasome formation during infection with *Anaplasma phagocytophilum* in macrophages through targeting caspase-1 activity [36].

In order to understand how Sialo L2, a tick salivary molecule, can support *Borrelia* establishment in the host, we studied the effect of tick cystatins on DC maturation and function. The effect on the production of chemokines, IFN-β and signalling pathways activated in dendritic cells by *Borrelia* spirochetes and relevant TLR ligands was analysed.

## Methods

### Animals

Female C57BL/6 mice (10 weeks of age) were obtained from Charles River Laboratories. All experiments were performed with permission from Local animal ethics committee of the Institute of Parasitology, Biology Centre ASCR České Budějovice, PID 167/2011.

### Bacteria

The strain of *Borrelia burgdorferi* sensu stricto obtained from ATCC collection was grown in Barbour-Stoenner-Kelly-H (BSK-H) medium (Sigma) supplemented with 6 % rabbit serum at 34 °C. The fourth passage was used in the experiments.

### Preparation of recombinant cystatins

Recombinant cystatins Sialo L and Sialo L2 were expressed in *Escherichia coli* followed by purification of active protein, as previously described [31, 35]. LPS contamination was removed by Arvys Proteins using the detergent-based extraction method. The presence of endotoxin was estimated with a sensitive fluorescent-based endotoxin assay (Lonza Biologics) and was <3 x 10^−14^ endotoxin g/μg protein for both cystatins. The endotoxin level did not exceed 2 pg/ml during testing the effect of cystatins on DC.

### Generation of bone-marrow-derived dendritic cells

Bone-marrow derived conventional dendritic cells (DC) and plasmacytoid (pDC) dendritic cells were prepared as described before [20, 21], respectively, with minor modifications. Briefly, mice were sacrificed by cervical dislocation, intact femurs and tibias were removed, and bone marrow was harvested by repeated flushing with MEM. To derive conventional DC, bone marrow cells (10^6^/ml) were cultured for 7 days in 6-well plate in RPMI 1640 medium supplemented with 10 % FCS, 50 mM HEPES, 2 mM glutamine, 50 μM mercaptoethanol, penicillin, streptomycin, amphotericin B, and 30 ng/ml of recombinant mouse GM-CSF (Sigma-Aldrich). Half of the medium was replaced with the fresh medium on day 3 and 5. On day 7, non-adherent cells were harvested and used as immature DC.

To analyse the effects of cystatins on DC differentiation, 10^5^ bone-marrow cells were seeded in 96-well plate in the same medium as described above (including GM-CSF) and the Sialo L or Sialo L2 were added to the culture on day 3 to final concentration 3 μM. Cells were fed on day 5 and 7, and harvested on day 9. Surface expression of MHC class II was determined by flow cytometry within CD11c-positive population.

To derive plasmacytoid cells, bone marrow cells (1.5 × 10^6^ / ml) were cultured for 8 days in 6-well plate in RPMI 1640 medium supplemented with 10 % FCS, sodium pyruvate, glutamine, penicillin, streptomycin, amphotericin B (PAA) and 100 ng/ml of recombinant human Flt-3L (R&D Systems). Half of the medium was replaced once after 4 days of culture. On day 8, non-adherent cells were harvested, washed in fresh medium and used in subsequent experiments.

### IFN-β measurement

Freshly derived pDC were seeded in 96-well plate at a concentration of 2 × 10^5^ cells per well. Following 2 h incubation with Sialo L or Sialo L2 (each 3 μM) the cells were stimulated with spirochetes at MOI = 10 (10 spirochetes per 1 cell), imiquimod (R837, 2 μg/ml) (InvivoGen), or CpG (ODN1668, 50 nM) (Enzo Life Sciences). MOI = 10 was sufficient to activate DC as shown previously [37]. IFN-β was determined in cell-free culture supernatants harvested 5 and 16 h after stimulation using LEGEND MAX™ mouse IFN-β ELISA Kit (BioLegend) following the manufacturer’s instructions.

### Chemokine measurements

BMDC were seeded at concentration 0.5 × 10^6^ or 2 × 10^5^ cells per well in 24-well plate or 96-well plate, respectively. Next day DCs were incubated 2 h with Sialo L or Sialo L2 (both 3 μM) and then *B. burgdorferi* was added at MOI = 10. After 24 h, cell-free supernatants were collected and analysed in Proteome Profiler™ antibody array according the manufacturer’s instructions (R&D). The chemokines were visualized by enhanced chemiluminescence and the abundance of signal was measured using CCD image system (ChemiDoc™ MP Imaging System) and Image Lab software, v. 4.1 (BIO-RAD). Alternatively, the amount of secreted chemokines (IP-10, MPC-1, MIP-1α, MIP-1β, and MIP-2) was determined in cell-free culture supernatants using ELISA kits (PeproTech) following the manufacturer’s instructions.

### Flow cytometry

Bone marrow-derived pDC were seeded on 96-well plate at the concentration of 1 × 10^6^ cells per ml of complete culture medium with Flt-3L and pretreated with either Sialo L or Sialo L2 (both 3 μM). After 2 h, cells were activated either with imiquimod (2 μg/ml), CpG (ODN1668, 50 nM) or *B. burgdorferi* spirochetes (MOI = 10). After 24 h incubation, cells were washed once in PBS with 1 % FCS and stained for flow cytometry analysis with anti-CD11c-PE mAb, anti-MHCII-AlexaFluor700 mAb, anti-CD86-APC mAb (all from eBioscience), anti-CD11b-FITC mAb, and anti-B220-PE-Vio770 mAb (both from Miltenyi Biotech). Dead cells were excluded from analysis using propidium iodide. Flow cytometry was performed on FACS Canto II flow cytometer and data were analysed using FACS Diva software, v. 5.0 (BD Biosciences). Plasmacytoid DCs were gated from living single cells as CD11c+, CD11b- and B220+. Levels of expression of CD86 were measured as MFI of APC.

### Immunoblotting

BMDC were seeded at 0.5 × 10^6^ cells per well in 24-well plate. Next day DCs were incubated 2 h with tick cystatins (each 3 μM) prior to the addition of LTA (2 μg/ml) for 15, 30, and 60 min or *Borrelia* spirochetes (MOI = 10) for 15, 30, 60, and 120 min. Afterwards, cells were lysed in a RIPA buffer (1 % Nonidet P-40, 0.25 % sodium deoxycholate, 1 mM EGTA, 150 mM NaCl, and 50 mM Tris-HCl (pH 7.5)) in the presence of protease inhibitors (10 μg/ml aprotinin, 1 μg/ml leupeptin, 1 mM phenylmethylsulfonyl fluoride, 1 μg/ml pepstatin) and phosphatase inhibitors (25 mM sodium fluoride and 2 mM sodium orthovanadate). 20 μg of total proteins were separated by SDS-PAGE using an 8 % gel and then electro-transferred to Immobilon-P membranes. The blots were incubated overnight at 4 °C with the antibody recognizing phospho-NF-κB p65 (Ser^536^), phospho-p44/42 MAPK (Erk1/2) (Thr^202^/Tyr^204^), phospho-p38 MAPK (Thr^180^/Tyr^182^), phospho-Akt (Ser^473^), total NF-κB p65, p44/42 MAPK (Erk1/2), p38 MAPK, Akt, and β-actin (all from Cell Signalling) followed by incubation with secondary antibody conjungated with horse radish peroxidase. The proteins were visualized using enhanced chemiluminescence (Pierce), and their abundance was analysed using CCD image system (ChemiDoc™ MP Imaging System) and Image Lab software, v. 4.1 (BIO-RAD).

### Statistical analysis

One-way analysis of variance (ANOVA) followed by Bonferroni test in GraphPad Prism, version 5.0 was used to compare the differences between control and treated groups. P ≤ 0.05 was considered statistically significant and is marked by one star, P ≤ 0.01 is marked by two stars.

## Results

### Sialostatin L2 decreases the MIP-α and IP-10 production by dendritic cells in response to *Borrelia burgdorferi*

**Fig. 1:**
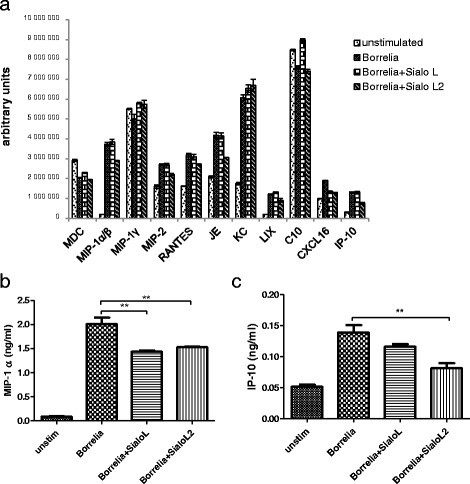
Sialostatin L2 inhibits the MIP-α and IP-10 production by dendritic cells in response to *Borrelia burgdorferi*. Dendritic cells were exposed to *B. burgdorferi* at MOI = 10 in the presence or absence of sialostatin L (3 μM) or sialostatin L2 (3 μM). After 24 h, supernatants were collected and used in proteome chemokine array. The chemokines were visualized by enhanced chemiluminescence and their amount is expressed as arbitrary units (**a**). Similarly, supernatants from cells treated as described above were analysed in ELISA assay for the presence of MIP-1α (**b**) and IP-10 (**c**). Graphs (in **b**, **c**) show results of one representative experiment out of at least three independent experiments performed

Numbers of chemokines known to recruit leukocytes to the infection site are upregulated in DC during *Borrelia* infection [18, 38]. We aimed to determine the effect of cystatins on chemokine production by bone-marrow derived dendritic cells upon *Borrelia* stimulation. We utilised proteome chemokine array to screen which chemokines are induced by *Borrelia* spirochetes, and which might be affected by sialostatins. Addition of borreliae resulted in a 3.5-fold increase of neutrophil-recruiting chemokine CXCL1 (KC), 4.5-fold rise of CXCL10 (IP-10) and 18.6-fold increase of monocyte/macrophage recruiting chemokine CCL3/CCL4 (MIP-1α/β). Two-fold rise and less was observed in case of CXCL2 (MIP-2), CCL5 (RANTES), CCL2 (MCP-1/JE), CXCL5 (LIX), and CXCL16 (Fig. 1a). The production of all tested chemokines was reduced by Sialo L2, except for KC and MIP-1γ which remained unchanged (MIP-1α/β by 23 %, MIP-2 by 18 %, RANTES by 15 %, JE by 29 %, LIX by 25 %, CXCL16 by 32 % and IP-10 by 44 %). Sialo L, in contrast to Sialo L2, did not influence either of these chemokines in the array. The effect of sialostatins on selected chemokines (MIP-1α, MIP-1β, IP-10, MIP-2, and JE) was further analysed by ELISA. The inhibitory effect of sialostatin L2 was confirmed for two chemokines; the production of MIP-1α and IP-10 was significantly decreased (Fig. 1b, c). However, we did not observe any effect of sialostatin L2 on other tested chemokines (data not shown). Interestingly, MIP-1α was inhibited also by sialostatin L. This was not seen in the proteome array likely because of using pan antibody not able to distinguish between MIP-1α and MIP-1β.

### The effect of sialostatin L2 on the signalling pathways activated by LTA and *Borrelia burgdorferi* in dendritic cells

**Fig. 2:**
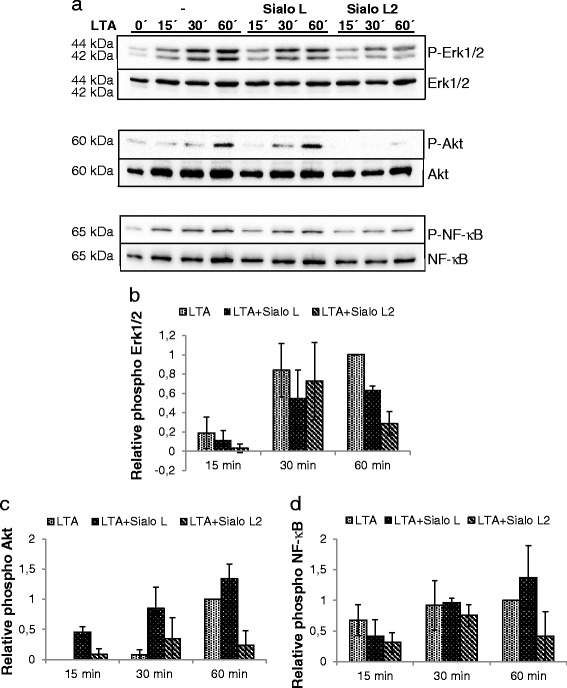
Effect of sialostatins on the signalling pathways activated by LTA in dendritic cells. Dendritic cells were seeded in 24-well plate. Next day DCs were incubated 2 h with tick cystatins (both 3 μM) prior to the addition of LTA (2 μg/ml) and further incubated for indicated times. Afterwards, cells were lysed and obtained protein extract was further analysed by immunoblotting using antibodies recognizing phosphorylated form of tested kinases. Membranes were stripped and reprobed with antibodies against total kinase protein, which served as a control (**a**). Proteins were visualized by enhanced chemiluminescence. Bands were quantified using scanning densitometry and phosphorylation/activities of Erk1/2 (**b**), Akt (**c**), and NF-κB (**d**) kinases were normalized by total kinase protein level (relative activity = phospho kinase/total kinase). Three independent experiments were performed and representative blots are shown. Graphs represent the average ± SD from 2-3 experiments, the relative phosphorylation of kinase achieved at 60 min upon LTA stimulation was set up to 1 to allow pooling of data

Induction of proinflammatory mediators by *B. burgdorferi* is mediated by multiple signalling pathways through ligation of several TLRs. Because TLR-2 is known to be strongly activated by *Borrelia* lipoproteins, we first tested the activation of signalling pathways upon addition of lipoteichoic acid (LTA), a ligand for TLR-2, in the presence or absence of both cystatins. The pathways important for induction of pro- inflammatory cytokines and chemokines were analysed: Erk1/2 and p38 MAP kinases, NF-κB, and PI3K/Akt pathways (Fig. 2a). Sialo L2 attenuated phosphorylation of Erk1/2 (decrease by 72 % at 60 min), while Sialo L decreased this signalling molecule by 37 % at 60 min (Fig. 2b). The activation of p38 MAP kinase remained unchanged in the presence of both cystatins (Additional file 1a). Interestingly, the activation of PI3K pathway, measured by the phosphorylation of its downstream target Akt, was decreased by 76 % in the presence of Sialo L2. No such effect was observed in case of Sialo L (Fig. 2c). The phosphorylation of NF-κB was decreased by sialostatin L2 by 59 % at 60 min (Fig. 2d).

**Fig. 3:**
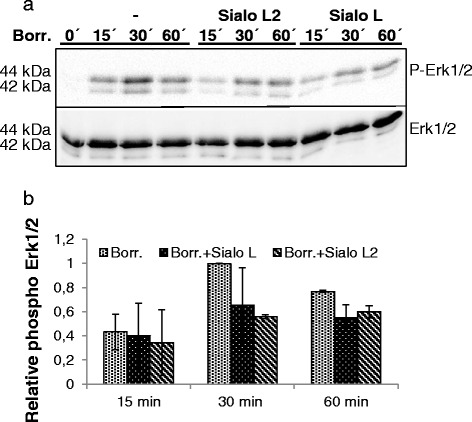
Effect of sialostatin L2 and sialostatin L on the Erk1/2 signalling pathway activated by *Borrelia burgdorferi* in dendritic cells. Dendritic cells were seeded in 24-well plate. Next day DCs were incubated 2 h with sialostatin L2 and sialostatin L (both 3 μM) prior to the addition of Borreliae (MOI = 10) and further incubated for indicated times. Afterwards, cells were lysed and obtained protein extract was analysed by western blotting using antibodies against phosphorylated form of Erk1/2 and total Erk1/2 (**a**). Proteins were visualized by enhanced chemiluminescence. Relative phosphorylation/activity of Erk1/2 (**b**) kinase is shown; signal corresponding to phosphorylated kinase was adjusted to total kinase level. Three independent experiments were performed with sialostatin L2 and two with sialostatin L and representative blots are shown. Graph represent the average ± SD from 2 experiments, the phosphorylation of kinase achieved at 30 min upon *Borrelia* addition was set up to 1 to allow pooling of data

Similarly, dendritic cells were exposed to *B. burgdorferi* and the effect of Sialo L2 and Sialo L on signalling pathways was analysed. All tested pathways were activated with different kinetics compared to LTA. The phosphorylation of Erk1/2 kinase was impaired by Sialo L2 (decrease by 45 % at 30 min and by 22 % at 60 min) and by Sialo L by 29 % at 60 min (Fig. 3a, b). The phosphorylation of p38 MAPK, NF- κB and Akt remained unaffected in the presence of both Sialo L2 and Sialo L (Additional file 1b).

### Sialostatin L decreases production of IFN-β in plasmacytoid dendritic cells activated by *Borrelia burgdorferi* and TLR-7 ligand

**Fig. 4:**
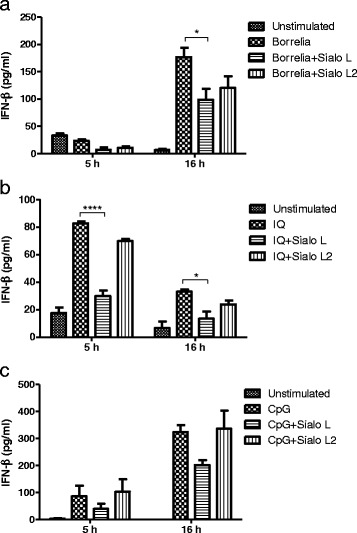
The effect of tick sialostatins on the production of IFN-β in plasmacytoid dendritic cells activated by *Borrelia*, TLR-7, and TLR-9 ligands. Plasmacytoid dendritic cells were activated with *B. burgdorferi* at MOI = 10 (**a**), imiquimod (2 μg/ml) (**b**) or CpG (50 nM) (**c**) in the presence or absence of sialostatins (both 3μM). Supernatants were collected 6 and 16 h after stimulation and analysed by ELISA assay

It has been shown that Borreliae are able to induce type I IFNs (IFN-α and IFN-β) in macrophages and dendritic cells and that this induction is mediated by endosomal TLR7/8 and TLR9 receptors [7–9]. Plasmacytoid (pDC) dendritic cells were chosen to examine the effect of tick cystatins on *Borrelia,* TLR-7, and TLR-9 - induced production of IFN-β. This subset of DC is known for great production of type I IFN and higher expression of TLR-7 and TLR-9 [39]. pDC were activated with *Borrelia* spirochetes, imiquimod (TLR-7 agonist), and CpG (TLR-9 agonist) in the presence or absence of cystatins and subsequently the amount of IFN-β was determined at indicated time points (chosen according to Petzke *et al.* [9] (Fig. 4). Upon addition of *Borrelia* spirochetes to cells we observed the induction of IFN-β mainly at later time point and this induction was significantly decreased by sialostatin L (Fig. 4a). Similarly, the amount of secreted IFN-β was significantly decreased by sialostatin L upon TLR-7 ligation at both tested time points (Fig. 4b). The presence of sialostatin L2 did not influence the amount of produced IFN-β. When pDC were stimulated with CpG, the production of IFN-β was more robust and increased with time. Sialostatin L decreased the IFN-β production by almost 50 % (without statistical significance), and sialostatin L2 remained without effect (Fig. 4c).

### Sialostatin L negatively affects TLR-7 and TLR-9 mediated maturation of DCs but does not influence *Borrelia burgdorferi* induced maturation

**Fig. 5:**
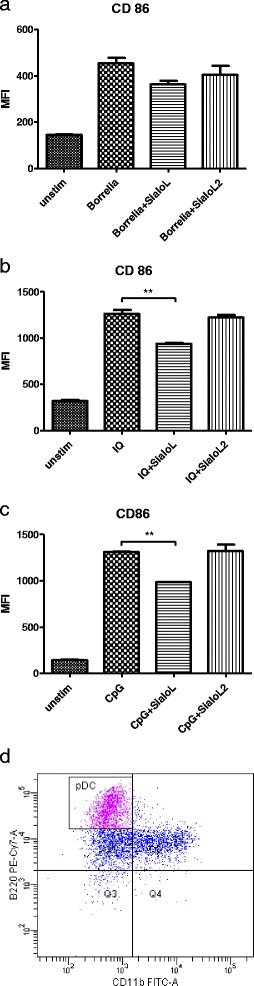
Maturation of plasmacytoid dendritic cells induced by *Borrelia*, TLR-7, and TLR-9 ligation in the presence of sialostatins. Plasmacytoid dendritic cells were activated with *Borrelia* at MOI = 10 (**a**), imiquimod (2 μg/ml) (**b**), or CpG (50 nM) (**c**) in the presence or absence of sialostatins (both 3 μM). The expression of costimulatory molecule CD86 was analysed by flow cytometry among CD11c+, CD11b-, and B220+ cell population. Medium fluorescence intensity (MFI) is shown. The phenotype of plasmacytoid DC from CD11c population is shown (**d**)

Dendritic cells, upon sensing pathogen, undergo process of maturation, which is accompanied by an increase of expression of some co-stimulatory molecules, like CD86, CD40, and CD80. We wondered whether cystatins Sialo L and Sialo L2 can influence the maturation of plasmacytoid dendritic cells stimulated by *B. burgdorferi*, TLR-7 and TLR-9 ligands. The expression of co-stimulatory molecule CD86 was analysed by flow cytometry. The phenotype of pDC is shown in Fig. 5d, pDC were gated as CD11c+, CD11b-, and B220+ cells. As expected, addition of *Borrelia* spirochetes led to the increase of CD86 expression (Fig. 5a). However, the expression of CD86 increased to the comparable levels also in the presence of tested cystatins. Thus *Borrelia*- induced maturation was not affected by cystatins. On the contrary, in imiquimod-stimulated pDC, was observed small but significant decrease in CD86 surface expression in the presence of sialostatin L compared to control (Fig. 5b). Similarly, the increase of CD86 expression on DC, induced by ligation of TLR-9, was inhibited by sialostatin L, but not sialostatin L2 (Fig. 5c). Thus sialostatin L negatively affects TLR-7, and TLR-9 mediated maturation of DC but does not significantly affect *Borrelia*-induced maturation.

### Sialostatin L reduces differentiation of bone-marrow DC

**Fig. 6:**
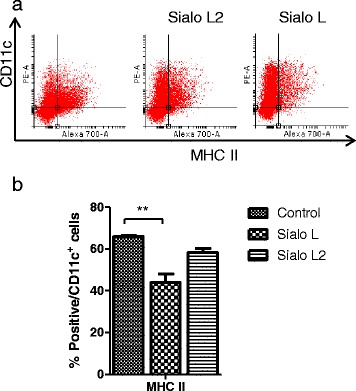
Differentiation of dendritic cells from bone-marrow cells is inhibited by sialostatin L. Bone-marrow cells were cultured in the presence of GM-CSF and on day 3 the Sialo L or Sialo L2 (3 μM) were added to cultures. After 8 days, cells were harvested and analysed by flow cytometry for surface expression of MHC class II and CD11c (**a**). Differentiated cells (MHC II and CD11c positive) are shown as a percentage of total CD11c positive cells (**b**). The representative of three independent experiments is shown

As salivary molecules have an opportunity to enter bone marrow through the bloodstream, we decided to examine the influence of cystatins on the differentiation of dendritic cells from bone-marrow cells. The experiment was performed according to Sun *et al.* [40]. Bone-marrow cells were cultured (differentiated) in the presence of GM-CSF and on day 3 sialostatin L or sialostatin L2 were added to the cultures. After 8 days, cells were harvested and the expression of MHC class II molecules was determined. As shown in Fig. 6, among the CD11c positive cells, the number of MHCII positive cells reached 65.85 %. In the presence of sialostatin L the number of MHCII - positive cells decreased significantly to 43.91 %. Sialo L2 did not affect significantly the percentage of MHC class II positive cells.

## Discussion

Sialo L2 and Sialo L are tick salivary cystatins, which are together with other salivary compounds released by the hard tick *I. scapularis* into the wound during tick feeding. During this process *B. burgdorferi* could be transmitted to the host. In response to *Borrelia* spirochetes, dendritic cells and other skin-resident immunocompetent cells become activated which leads to the production of proinflammatory mediators attracting further immune cells to the site of infection and activating them. These events can lead to clearing of most bacteria. It has been shown that Sialo L2, when injected intradermally into the mice, increased the burden of *Borrelia* spirochetes in the skin [35]. We hypothesized that observed effect could result from Sialo L2 evoked changes in dendritic cells function. Therefore we analysed the effect of Sialo L2 and related cystatin Sialo L on the immuno-modulatory function and signal transduction of mouse bone-marrow derived dendritic cells (DC) activated by *Borrelia* and relevant TLR ligands. We found that these two tick cystatins differentially modulate the function of DC. While Sialo L2 inhibited the production of chemokines MIP-1α and IP-10 in response to *Borrelia* spirochetes and attenuated the activation of Erk1/2, PI3K/Akt, and NF-κB pathways in response to TLR-2 ligation (the major receptor activated by spirochetal lipoproteins), the related cystatin Sialo L suppressed the production of IFN-β and attenuated the maturation and differentiation of DC.

In our *ex vivo* experiments, *Borrelia-*stimulated bone-marrow dendritic cells secreted several chemokines, including neutrophil-, monocyte/macrophage-, and T cell-recruiting chemokines, similarly as was reported by other studies [18, 38]. Sialo L2 suppressed significantly production of two chemokines, MIP-1α and IP-10. MIP-1α is a chemotactic factor for mononuclear cells, T cells, and mast cells and plays a role in differentiation of type 1 Th lymphocytes. IP-10 is a CXC chemokine and attracts, in addition to monocytes and Th1 cells, also NK cells [41]. We predict that the recruitment of these cells could be impaired by Sialo L2 *in vivo*.

Dendritic cells are among the first immune cells to come into contact with *Borrelia* in the skin [1]. Phagocytosis of *Borrelia* spirochetes leads to production of various proinflammatory cytokines [42] including chemokines. Inhibitory effect of sialostatin L2 on the production of chemokines attracting inflammatory cells into tick feeding site can lead to reduced inflammation due to tick saliva effect [43]. Reduced influx of inflammatory cells could facilitate establishment and proliferation of spirochetes in the skin [44].

Dendritic cells are equipped with several pattern recognition receptors (PRR), which sense Borreliae, including TLR, NLR, and LTR [2]. To reveal the mechanism of Sialo L2 effect on chemokine production by *Borrelia*-activated DC, we analysed the activation of chosen signalling molecules first upon TLR-2 ligation. TLR-2 is robustly activated by *Borrelia* lipoproteins [5] and critically involved in production of pro-inflammatory mediators, including chemokines. Moreover, TLR have an essential role in the control of *B. burgdorferi* burden [2, 4], which is enhanced by Sialo L2 *in vivo* [35]. The most pronounced effect of Sialo L2 on activation of tested signalling molecules in response to LTA was observed on phosphorylation of Akt, the downstream target of PI3K pathway. Interestingly, even the basal level of this kinase was decreased by Sialo L2. Consequences of PI3K pathway inhibition can be predicted. The inhibition of PI3K significantly impaired induction of chemokine and cytokine genes via TLR-2 in DC, including IP-10 [45]. Of note, PI3K pathway plays an important role in phagocytosis of *Borrelia* spirochetes by macrophages [14]. The inhibition of Akt phosphorylation was not observed by Sialo L2 in *Borrelia*-activated DC, possibly due to weak activation of this kinase.

The other pathway attenuated by Sialo L2 (in LTA and *Borrelia* activated DC) was Erk1/2 mediated cascade. Both, Erk1/2 and PI3K kinases are indispensable for induction of MIP-1α and MCP-1 in LTA stimulated murine macrophages [46]. IP-10 induction is mediated by IFNs (often produced in response to microbial products) and its upregulation is associated with the activation of JAK1, JAK2/STAT1 and MAPK pathways [47–49]. The decline of MIP-1α and IP-10 production in *Borrelia*-activated DCs by Sialo L2 could be thus mediated via inhibition of the Erk1/2 and PI3K signalling pathways. Recently, we have found that Sialo L2 attenuates IFN signalling triggered by IFN-β or LPS which leads to the suppression of interferon stimulated genes like IRF-7 and IP-10 [50]. The decrease of IP-10 production by Sialo L2 in response to *Borrelia* spirochetes could be in part also a consequence of impaired IFN/JAK/STAT signalling.

The third pathway influenced by sialostatin L2 upon LTA stimulation was NF-κB pathway. The involvement of NF-κB pathway in the induction of proinflammatory mediators was documented; e.g. TLR-2/NF-κB/MAPK signalling plays a key role in IL-8 induction in macrophage cell line THP-1 exposed to *B. burgdorferi* [51]. We however did not detect any defect in the activation of this pathway in response to borreliae. Since dendritic cells sense borreliae by several PRR [2], the moderate effect of Sialo L2 on signals triggered through TLR-2 could be masked by signals triggered through other receptors.

In addition to chemokines, type I interferons are important cytokines modulating immune response to pathogens. *B. burgdorferi* is able to induce type I IFN and this induction is mediated through endosomal receptors TLR-7 and TLR-9 [6–9]. Plasmacytoid DC are major producers of type I IFN [52]. We found out that in plasmacytoid dendritic cells, the amount of produced IFN-β in response to *Borrelia* spirochetes and TLR-7 activation was decreased by sialostatin L and only weakly or not at all by sialostatin L2. IFN is pleotropic cytokine which recruits NK cells, has a direct antiviral effect on cells, and links the innate and adaptive immunity.

The down-regulation of IFN-β production by Sialo L in *Borrelia*/TLR-7/TLR-9 stimulated cells may have further consequences for the development of adaptive immune responses. In general, type I interferon directly influences the fate of CD4+ and CD8+ T cells during the initial phases of antigen recognition contributing to Th1 commitment and negatively regulating Th2 and Th17 differentiation [53]. Down-regulation of interferon can bring about an opposite effect. Moreover as sialostatin L inhibits production of IL-12 and TNF-α by DC as well as their differentiation [30], it probably leads to Th2 polarization of the immune response which is advantageous for *Borrelia* establishment in the skin [54]. In addition to modulation of the Th differentiation, type I IFNs also positively influence DC maturation [55, 56].

Indeed, we show that the maturation of plasmacytoid DC induced by TLR-7 or TLR-9 ligands was also decreased by Sialo L (judged by expression of co-stimulatory molecule CD86). When the maturation of DC was initiated by borreliae, only statistically not significant decline in CD86 expression was observed in the presence of Sialo L, presumably due to the fact that *Borrelia* spirochetes are weaker inducers of maturation then TLR ligands. In agreement, it was previously published that Sialo L inhibits the maturation of DC induced by LPS; it negatively affects the expression of the costimulatory molecules CD80 and CD86 [30]. Thus, Sialo L influenced function of dendritic cells in a different way in comparison to Sialo L2.

We did not investigate the mechanism which is behind the declined IFN-β production due to sialostatin L effect. However, since cathepsin L has been implicated in processing of TLR-9 [57], and sialostatins L and L2 are strong inhibitors of this protease [35], we could speculate that the decline of IFN-β is the result of impaired TLR-9 processing. Moreover, the amount of endogenously produced IFN-β was not affected by sialostatins in splenic DCs stimulated with TLR-4 agonist, where no processing had occurred [50].

Finally we examined the effect of tick cystatins on the differentiation/derivation of dendritic cells from bone marrow and found that Sialo L negatively affects the number of differentiated dendritic cells (MHC class II and CD11c positive cells). MHC class II molecule is necessary for the presentation of antigen to naive T-cells. As cathepsin S is implicated in the processing of the invariant chain within MHC class II antigens and sialostatin L strongly inhibits this protease [30], it seems likely that the decrease in MHC class II expression is mediated through inhibition of cathepsin S [58]. The inhibitory effect on differentiation of BMDC (measured by expression of MHC class II molecules) was also reported for cystatin rHp-CPI from murine nematode parasite *Heligmosomoides polygyrus* [40].

## Conclusions

We show here that two related tick sialostatins affect different functions of dendritic cells. While sialostatin L influences the maturation of DC in part through the inhibition of IFN-β having thus an impact on adaptive immune response, sialostatin L2 affects, through attenuation of several signalling pathways, the production of chemokines engaged in the development of inflammation.

## Additional file

Additional file 1: **Effect of sialostatins on the signalling pathways activated by LTA and** ***Borrelia burgdorferi*** **in dendritic cells.** Dendritic cells were seeded in 24-well plate. Next day DCs were incubated 2 h with tick cystatins (both 3 μM) prior to the addition of LTA (2 μg/ml) or Borreliae (MOI = 10) and further incubated for indicated times. Afterwards, cells were lysed and obtained protein extract was further analysed by immunoblotting using antibodies recognizing phosphorylated form of tested kinases. Afterwards, membranes were reprobed with antibodies against total kinase protein (a) or β-actin (b) which served as a control. Proteins were visualized by enhanced chemiluminescence.
