# Supplementary figures and images for: Tick sialostatins L and L2 differentially influence dendritic cell responses to Borrelia spirochetes
Source: Parasit Vectors. 2015 May 15;8:275. doi: 10.1186/s13071-015-0887-1 (PMC4436792; doi:10.1186/s13071-015-0887-1)

a

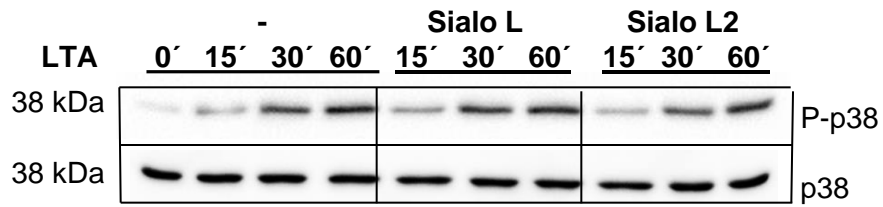

b

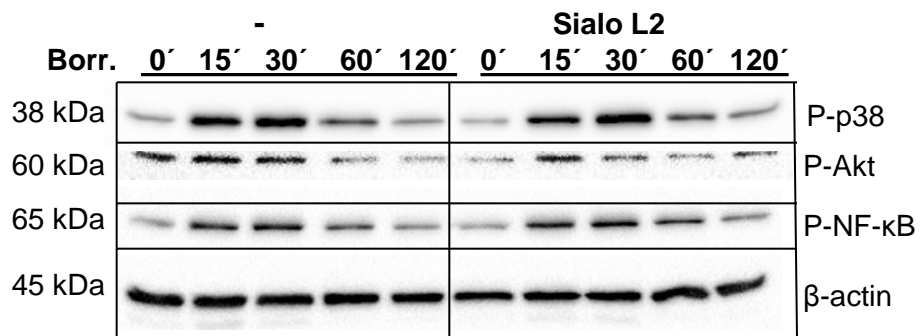

c

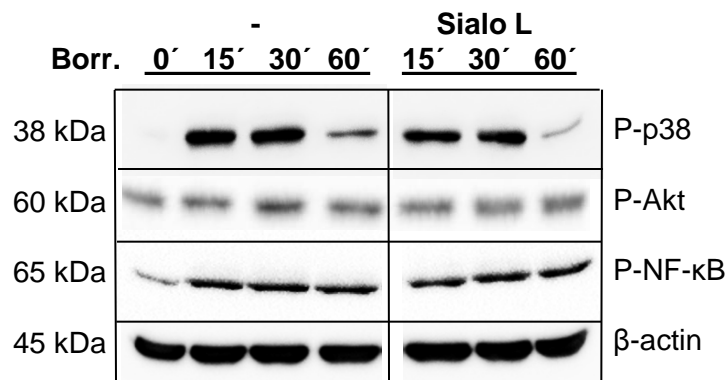

Supplement: Supplementary file 1 — Effect of sialostatins on the signalling pathways activated by LTA and Borrelia burgdorferi in dendritic cells. Dendritic cells were seeded in 24-well plate. Next day DCs were incubated 2 h with tick cystatins (both 3 μM) prior to the addition of LTA (2 μg/ml) or Borreliae (MOI = 10) and further incubated for indicated times. Afterwards, cells were lysed and obtained protein extract was further analysed by immunoblotting using antibodies recognizing phosphorylated form of tested kinases. Afterwards, membranes were reprobed with antibodies against total kinase protein (a) or β-actin (b) which served as a control. Proteins were visualized by enhanced chemiluminescence. [file 13071_2015_887_MOESM1_ESM.zip › 13071_2015_887_add(a).pdf]

a

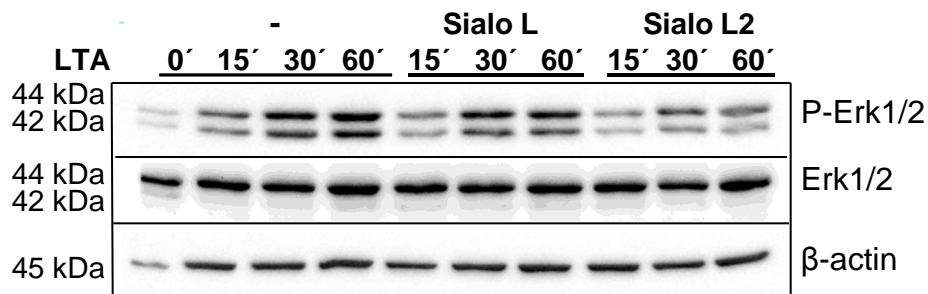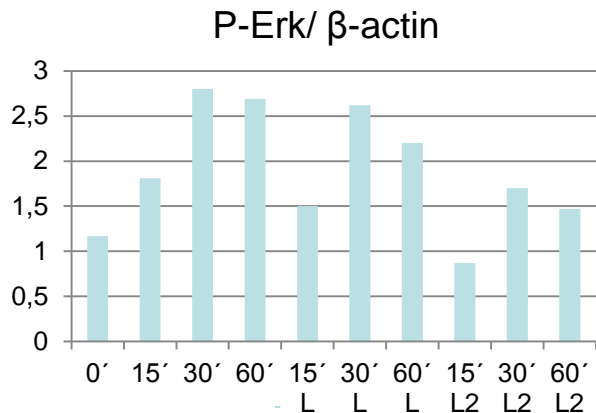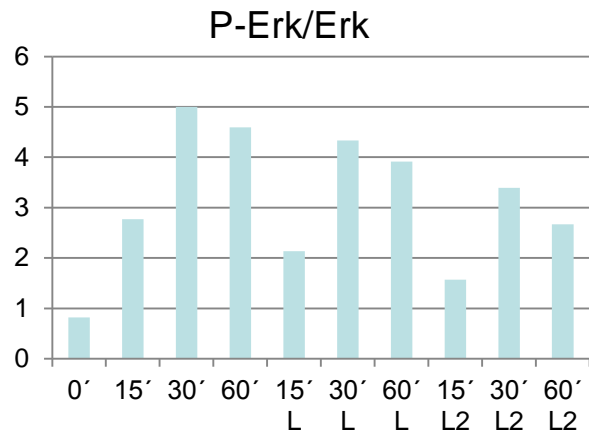

b

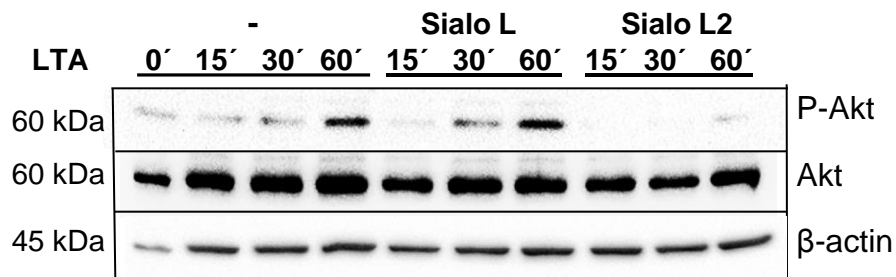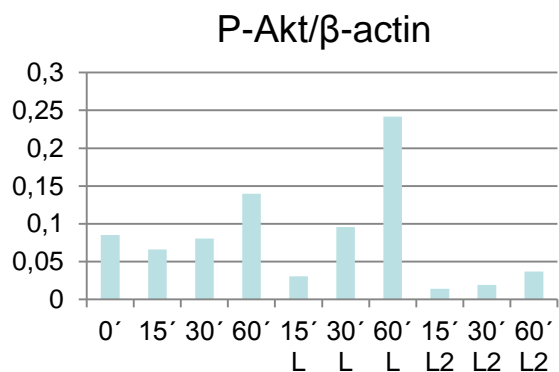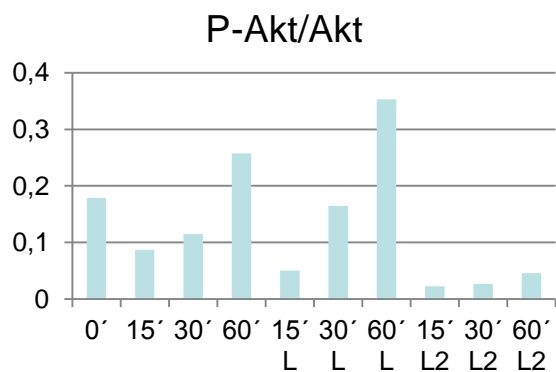

C

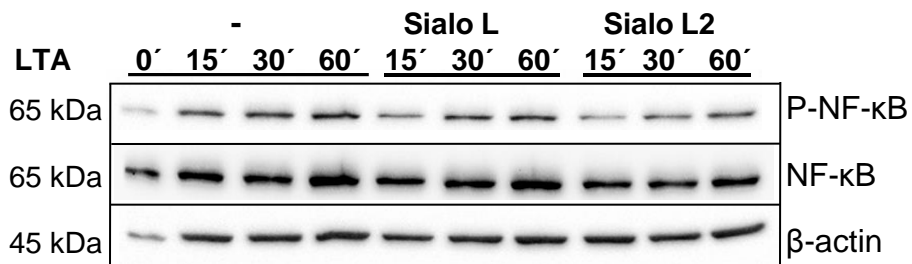

P-NF-κB/β-actin

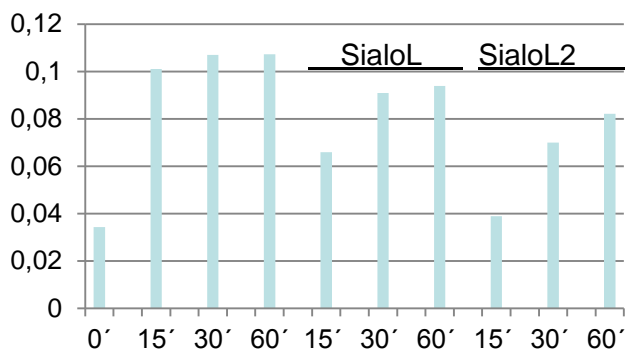

P-NF- κB/NF- κB

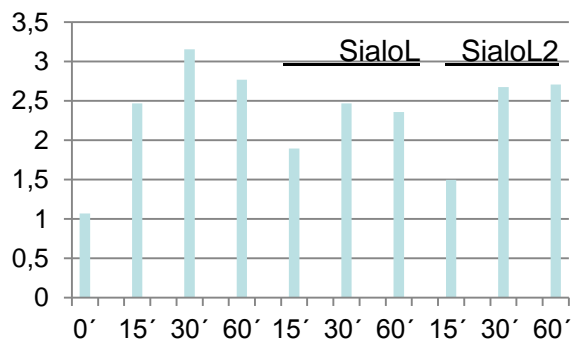

Supplement: Supplementary file 1 — Effect of sialostatins on the signalling pathways activated by LTA and Borrelia burgdorferi in dendritic cells. Dendritic cells were seeded in 24-well plate. Next day DCs were incubated 2 h with tick cystatins (both 3 μM) prior to the addition of LTA (2 μg/ml) or Borreliae (MOI = 10) and further incubated for indicated times. Afterwards, cells were lysed and obtained protein extract was further analysed by immunoblotting using antibodies recognizing phosphorylated form of tested kinases. Afterwards, membranes were reprobed with antibodies against total kinase protein (a) or β-actin (b) which served as a control. Proteins were visualized by enhanced chemiluminescence. [file 13071_2015_887_MOESM1_ESM.zip › 13071_2015_887_add(b).pdf]
